# Screw Coating as a Solution to Solve Screw Loosening Complications: An In Vitro Study

**DOI:** 10.3390/ma18122921

**Published:** 2025-06-19

**Authors:** Lara Coelho, Maria-Cristina Manzanares-Céspedes, Joana Mendes, Carlos Aroso, José Manuel Mendes

**Affiliations:** 1 Oral Pathology and Rehabilitation Research Unit (UNIPRO), University Institute of Health Sciences (IUCS), CESPU, 4585-116 Gandra, Portugal; mcmanzanares@ub.edu (M.-C.M.-C.); joana.silva.mendes@iucs.cespu.pt (J.M.); carlos.ribeiro@iucs.cespu.pt (C.A.); jose.mendes@iucs.cespu.pt (J.M.M.); 2 Human Anatomy and Embryology Unit, Faculty of Medicine and Health Sciences, University of Barcelona, 08007 Barcelona, Spain

**Keywords:** dental implants, lubricants, polytetrafluoroethylene, silicones, torque

## Abstract

**Background:** This study aimed to evaluate the influence of a screw coating on the screw preload and removal torque value (RTV) with and without the application of a cyclic load (CL) to make screws with greater untightening resistance to prevent screw loosening. **Methods**: Ninety complexes composed of implants, abutments, and prosthetic screws were examined and tested under CL oral conditions (*n =* 45) and non-CL conditions (nCL, *n =* 45). Each group was divided into three subgroups (*n* = 15): a control group (CG) without a screw coating, a GapSeal^®^-coated screw group (GG), and a polytetrafluoroethylene (PTFE) tape-wrapped screw group (PG). All screws were tightened at 30 Ncm, and the preload was recorded. In the nCL group, the screws were untightened to record the RTV. In the CL group, the screws were tightened, subjected to a CL in distillated water at a temperature of 37 °C, and then untightened to record the RTV. Micro-Ct analysis was conducted on two samples from each group before CL. SEM analyses of two samples per subgroup before and after CL were also performed. **Results**: The preload in the PG was significantly lower under nCL (29.92 Ncm) compared with CG (30.95 Ncm) and GG (31.19 Ncm) and also under a CL (PG: 30.92 Ncm) compared with CG (31.72 Ncm) and GG (31.42 Ncm). The RTVs of the PG were significantly lower under nCL (15.30 Ncm) compared with CG (27.98 Ncm) and GG (28.46 Ncm). Under CL, the RTVs of the PG were significantly higher (31.50 Ncm) compared with CG (26.00 Ncm) and GG (27.44 Ncm). **Conclusions**: Wrapping the screw with PTFE tape significantly reduced the preload but resulted in a significantly greater RTV under CL conditions in the simulated oral environment, suggesting that this could be a solution to decrease the risk of screw loosening.

## 1. Introduction

Currently, the main type of oral rehabilitation is the use of single crowns attached to dental implants. This type of treatment has been associated with high success rates for implants, ranging from 95.6 to 97.6%, and crowns attached to implants, ranging from 94.5 to 95.6% [1]. Despite the high success rate of this type of rehabilitation, several complications have been reported, with the loosening of the prosthetic screw being the most common complication, with an incidence between 4.6% and 12.7% [2,3].

When the prosthetic screw loosens, the preload, which is the initial load on the screw, is lost; however, this value should be maintained and fluctuate as little as possible [4]. Some factors that can contribute to the reduction in screw preload include external implant-abutment connections, dental implant single crowns, the presence of a cantilever, parafunctional habits, nonaxial loads, and the settling effect [5,6,7]. The settling effect plays a critical role in screw stability and is the result of the screw surface not being completely smooth. In addition, some areas of the screw are not in close contact with the internal area of the implant; in this case, settling occurs as the rough spots flatten out, which may be the main cause of screw loosening [8]. Siamos et al. [4] reported that approximately 2–10% of the initial preload is lost owing to the settling effect, which explains why the torque required to remove the crown—the removal torque value (RTV)—is lower than the torque initially used to tighten the screw. They also reported that the magnitude of the settling effect is directly correlated with the surface roughness and hardness, as well as the magnitude of the applied torque. Increasing the surface roughness results in an increase in the coefficient of friction and a decrease in the preload. To reduce this surface roughness, tightening techniques, such as retightening the prosthetic screws 10 min after the initial application of the torque and retightening the screw multiple times, have been proposed [4,9].

Coating is a means of incorporating a thin layer of material into a substrate (liquid or solid) by deposition. Different screw coating technologies, such as physical or chemical vapor deposition, plasma, and thermal spray, allow for the coating of screws with tungsten carbide [10], diamond-like carbon (DLC) [11], and pure gold [12]. These coatings act like lubricants to reduce the friction coefficient and increase the preload on the screw [13,14]. Due to the good results these screws afford, manufacturers began to sell prosthetic screws coated with some of these lubricants; nevertheless, they continue to loosen. Several authors have therefore begun to test coatings that can be applied during dental appointments, like saliva [15], Vaseline [16], blood, chlorhexidine (CHX), fluoride [17], silicone sealing gel [18], and polytetrafluoroethylene (PTFE) tape [19]. However, in addition to contradictory results, which are partly due to differences in the methodologies applied, some of these coatings are not applicable in the dental appointment, in which the loosening of prosthetic screws is a key concern. Adhesives have also been tested with excellent results; however, their biocompatibility is lacking, and their use is associated with an RTV that is much higher than the tightening torque, which may not allow for the removal of the crown if necessary, and thus, adhesives cannot be recommended [20].

Although several studies have tested different screw coatings to prevent screw loosening, this complication is still one of the most common, leading to a continued search for new solutions to solve this problem.

This study aimed to evaluate the influence of the application of two screw coating materials that can be applied during an appointment (PTFE tape and silicone sealing gel) on screw preload and RTV with and without the application of a CL to single crowns of dental implants. The null hypothesis was that coating the prosthetic screw with PTFE tape and silicone sealing gel would not affect the screw preload or the RTV. After applying the coating, to ensure that it did not create gaps in the interface between the abutment and the implant, we analyzed it using micro-computed tomography (Micro-CT), a METROTOM 1500 microtomograph (ZEISS, Oberkochen, Germany). A Scanning Electron Microscope (SEM), a QUANTA FEI 200 FEG-ESEM (FEI Company, Hillsboro, Oregon, USA) was used to analyze the untightened screws to determine the status of the coatings on the screw. If a coated screw significantly reduced the coefficient of friction, increased the screw preload, and increased the RTV, it could help prevent the loss of the prosthetic screw.

## 2. Materials and Methods

### 2.1. Sample Size Calculation and Preparation

**Figure 1:**
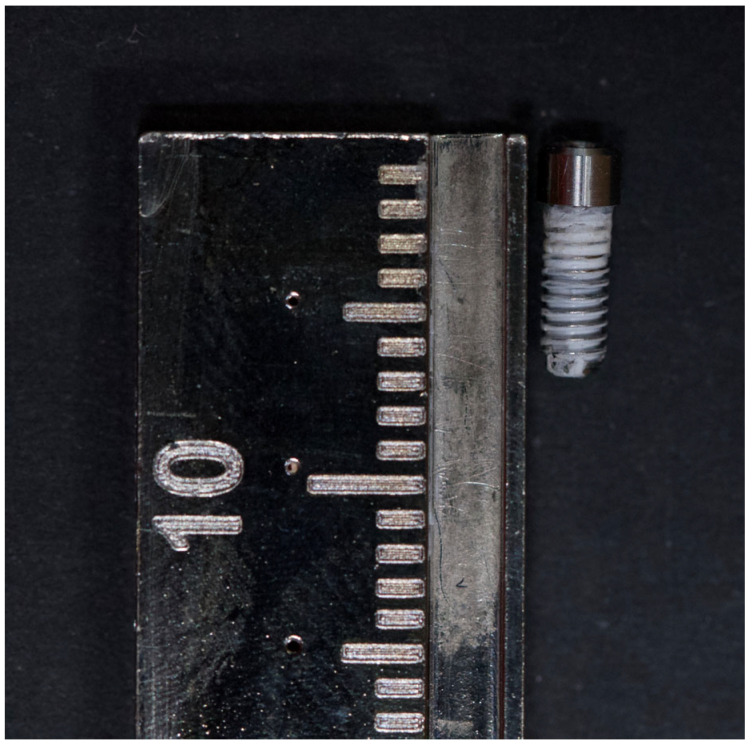
Prosthetic screw wrapped with PTFE tape.

The minimum sample size calculated for each group was 14, considering a minimum effect size of 0.5, β (power) = 0.80, and α (significance) = 0.05. In total, 90 complexes were examined, consisting of 90 external hex implants (length, 11.5 mm; diameter, 4.0 mm), 90 straight hex prosthetic abutments (sleeve height, 1 mm; length, 7 mm), and 90 original titanium prosthetic screws (Torx 1.7). All the components were obtained from DIU^®^ IMPLANT (Busan, Republic of Korea). The complexes were randomly and equally divided into two groups: Group 1 (*n =* 45), which was tested under noncyclic loading (nCL) conditions, and Group 2 (*n =* 45), which was tested under cyclic loading (CL) conditions. In each group, all the components were divided randomly and equally into three groups. The control group (CG; *n =* 15) included samples without any coating. In the GapSeal^®^ group (GG; *n* = 15), is a highly viscous silicone matrix with thymol, a biologically safe silicone sealing gel commonly used to prevent peri-implantitis [21,22], GapSeal^®^ (Lot: 01/14; Hager & Werken GmbH & Co., Duisburg, Germany), was injected into the internal compartment of the implants. In the PTFE group (PG; *n* = 15), the abutment screws were wrapped with PTFE tape (COD 269; 12 mm wide and 0.075 mm thick; MIARCO^®^, Valencia, Spain), which was wrapped twice around the screws; the PTFE tape edge was put in contact with the screw and the digital torque meter (iSD900 with CE0197; NSK^®^ Dental Spain SA, Madrid, Spain) was activated, until the PTFE tape wrapped the screw twice and then the PTFE tape was cut off at the bottom of the screw (Figure 1). PTFE is a biocompatible material used for other purposes in dentistry and in oral rehabilitation with implants, and PTFE tape is the current choice for screw access channel filling [23,24,25]. Initially, all the screws were manually screwed to the implant; then, the fit between the abutment and the implant was visually analyzed and we manually tested whether there was the presence of micromovements. Finally, the complex was coded with the name of the group and the sample. All tests were performed by a single-blinded trained operator with 25 years of experience in oral rehabilitation of dental implants.

**Figure 2:**
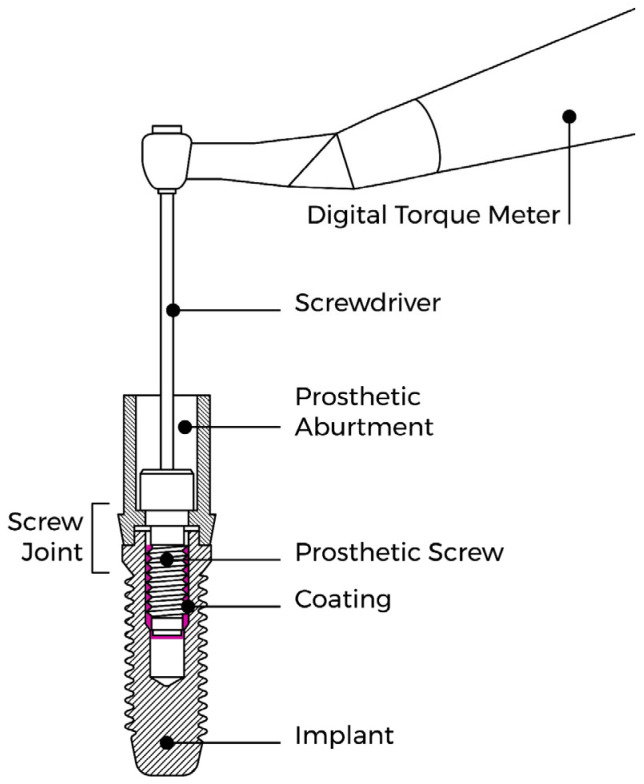
Representative diagram of implant complex, prosthetic abutment, prosthetic screw, screw joint, coating, digital torque meter, and screwdriver.

A representative diagram of the process can be found in Figure 2.

### 2.2. Measurement of the Screw Preload and RTV

**Figure 3:**
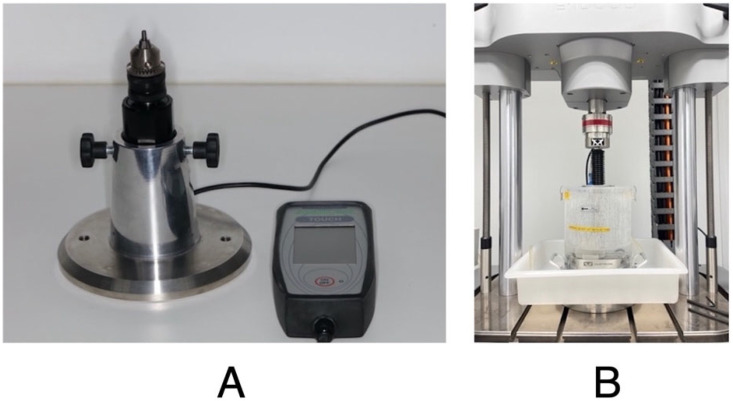
(**A**) Sample fixed in Centor Touch Star TH^®^ device and (**B**) sample fixed in Instron^®^ Electropuls E10000 LT testing machine for cyclic loading tests.

The screw preload and RTV were measured using a Centor Touch Star TH^®^ advanced touch screen dynamometer (Andilog Technologies, Vitrolles, France). Caligraph^®^ version 12.20 (Andilog Technologies, Vitrolles, France) was used for data acquisition. Data were continuously acquired at a frequency of 1000 Hz using a computer (Figure 3A). Each sample was tightened to 30 Ncm, as recommended by the manufacturer, using a screwdriver and a digital torque meter (iSD900 with CE0197; NSK^®^ Dental Spain SA, Madrid, Spain) calibrated during each measurement. After the screws were tightened and retightened for 10 min, as recommended by some authors [4,9], a dynamometer was used to record the preload generated by the screws, and in the nCL groups, the RTVs of the loosened screws were recorded. For the CL groups, the RTV was measured after performing the CL tests.

### 2.3. Cyclic Loading (CL) Tests

These experiments were performed using an Instron^®^ (Instron, Norwood, MA, USA) Electropuls E10000 LT testing machine with a Dynacell 2527 series ±250 N load cell. All test results were recorded using WaveMatrix TM2 version 2.0 test software (Instron^®^ Norwood, MA, USA). In these experiments, the samples were subjected to CL conditions following ISO 14801:2016 [26]. The samples were placed in an implant fixture block that ensured that the implant-abutment samples were mounted flush so that only the abutment protruded at an inclination angle of 30° with respect to the applied load. Distilled water was added to simulate the oral environment, and the temperature was maintained at 37 °C (Figure 3B). A dynamic sinusoidal load of 20–200 N was applied to the samples at a frequency of 2 Hz [26]. Each sample was subjected to 300,000 cycles of chewing force, simulating one year of chewing [19]. Approximately 42 h were required to test each sample. At the end of each test, the screws were untightened and the RTV was recorded.

### 2.4. Micro-CT Evaluation

Micro-CT analysis was performed on 2 samples of each in Group 1 (nCL). The samples underwent X-ray microtomography using a METROTOM 1500 microtomograph (ZEISS, Oberkochen, Germany) at a resolution of 10 μm, with a 360° angular rotation, a spacing between images of 0.2°, and three images were acquired per angle. After the complete scanning of the samples, the reconstruction of the images was carried out with VGL software version 3.4 (Volume Graphics GmbH, Heidelberg, Germany), adjusting the alignment of the sample and the artifacts. The same software was used to analyze the microgaps between the abutment and the implant. The implant/abutment complex was rotated 360 degrees, and the interface between the implant and the abutment was analyzed. The presence or absence of microgaps in all areas of the implant–abutment interface was recorded. If a microgap was present, it was measured in μm.

### 2.5. SEM Evaluation

**Figure 4:**
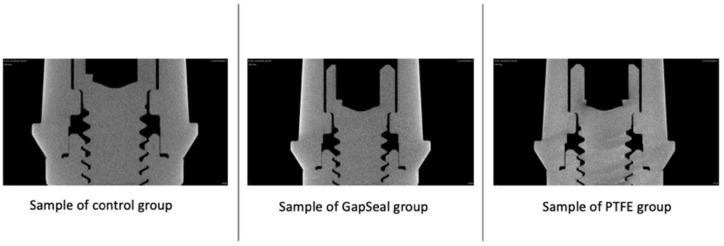
Micro-CT images of Group 1 (nCL) from control Group, GapSeal^®^ Group, and PTFE Group.

**Figure 5:**
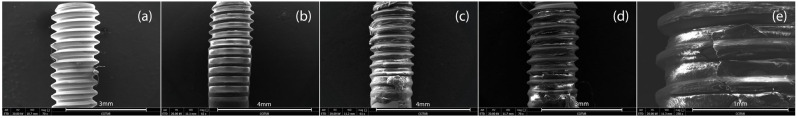
SEM images of Group 1 (nCL) (**a**) CG screw, (**b**) GG screw, (**c**) and (**d**) PG screws showing unequal distribution of PTFE tape, (**e**) close-up view of PG screw, in which it is possible to observe tears and cuts on PTFE tape.

The SEM analysis was performed by using a QUANTA FEI 200 FEG-ESEM (FEI Company, Hillsboro, Oregon, USA) Scanning Electron Microscope at 20kV. All the loaded and unloaded screws were loosened and observed directly to determine the status of the sealing gel and PTFE tape. The screws were affixed to mounting plates with carbon tape. The entire surface was viewed between ×60 to ×70, with some zones viewed at 253×. Of the 45 nCL samples, after loosening the screw and recording the RTV, 2 samples from each group were included for analysis using the SEM (Figure 4). Of the 45 CL samples, after the CL had ended, the screws were loosened to record the RTV, and 2 samples from each group were analyzed using the SEM (Figure 5). In total, 12 samples were analyzed in the SEM.

### 2.6. Statistical Analysis

The data were analyzed using R version 4.3.0 (R Core Team, 2023) [27]. Descriptive statistics are presented as the mean (M) and standard deviation (SD) for univariate analysis and the adjusted mean (adjM) and standard error (SE) for multivariate analysis. ANOVA (one-way analysis of variance) was used to compare the RTV between the groups. The effect size was calculated as eta squared (η^2^) for ANOVA. The thresholds were 0.01, 0.06, and 0.14, representing small, medium, and large effects, respectively. The level of significance was represented by *p*. The Shapiro-Wilk test was used to assess the normality of the distributions, and *p* < 0.05 was considered to indicate statistical significance. Levene’s test was used to assess the homogeneity of the variance, which was confirmed at *p* > 0.05. Multiple comparison tests were performed with Tukey’s HSD test. The significance threshold was set at 0.05.

For better understanding, a factorial plan was established (Table 1):

## 3. Results

### 3.1. Results for Group 1 (nCL)

The highest preload value was found in the GG (M = 31.19 Ncm, SD = 1.22), followed by the CG (M = 30.95 Ncm, SD = 1.00) and, finally, the PG (M = 29.92 Ncm, SD = 0.76). There were significant differences in the values of preload among the groups (CG, GG, and GP), F_(2, 42)_ = 6.56 [*p* = 0.003], η^2^ = 0.24, with a large effect size. Tukey’s multiple comparisons tests revealed significant differences in preload between the PG and GG (*p* = 0.004) and between the CG and PG (*p* = 0.022) (Table 2). Wrapping the screw with PTFE tape significantly reduced the preload in the nCL group.

The highest RTV was obtained in the GG (M = 28.48 Ncm, SD = 1.47), followed by the CG (M = 27.98 Ncm, SD = 1.20), and finally the PG (M = 15.30 Ncm, SD = 1.21). Univariate group comparisons for the RTV revealed statistically significant differences among the groups, with a high effect size (F _(2, 42)_ = 496.50 [*p* < 0.001], η^2^ = 0.94). Tukey’s multiple comparisons tests revealed significant differences in the RTV between the CG and PG (*p* < 0.001) and between the PG and GG (*p* < 0.001), but not between the CG and GG (*p* = 0.547) (Table 2). Wrapping the screw with PTFE tape significantly reduced the RTV in the nCL group.

### 3.2. Results for Group 2 (CL)

There were significant differences among the groups with respect to the preload and RTV. The highest preload value was found in the CG (M = 31.72 Ncm, SD = 1.16), followed by the GG (M = 31.42 Ncm, SD = 0.98) and, finally, the PG (M = 30.29 Ncm, SD = 0.83). The highest RTV was found in the PG (M = 31.50 Ncm, SD = 0.80), followed by the GG (M = 27.44 Ncm, SD = 3.76) and, finally, the CG (M = 26.00 Ncm, SD = 2.00). Furthermore, the preload and RTV were significantly associated with the CG, GG, and PG (F_(2, 42)_ = 8.51 [*p* < 0.001], η^2^ = 0.29; F_(2, 42)_ = 19.43 [*p* < 0.001], η^2^ = 0.48, respectively, with high effect sizes. Tukey’s multiple comparisons tests revealed significant differences between the PG and the GG and between the PG and the CG for all the variables: preload (*p* < 0.001 and *p* = 0.009), RTV (*p* < 0.001 and *p* < 0.001) (Table 3). Wrapping the screw with PTFE tape significantly reduced the preload, but after CL, it significantly increased the RTV.

### 3.3. Results for Micro-CT Analysis

A total of six samples were evaluated via Micro-CT, with two samples taken from each group (CG, GG, and PG). The images resulting from the Micro-CT were reconstructed, and an analysis of the presence or absence of microgaps between the abutments and the implants was performed. Micro-CT images revealed no microgaps (Figure 4). We can see that the application of these coatings did not influence the fit of the abutment with the implant. 

### 3.4. Results for SEM Analysis Results

**Figure 6:**
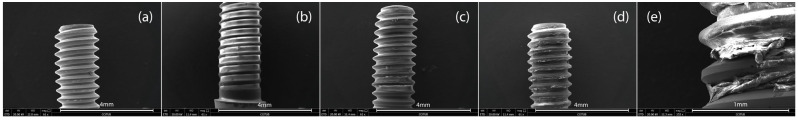
SEM images of Group 2 (CL) (**a**) CG screws (**b**) and (**c**) GG screw, showing an unequal distribution of the GapSeal^®^, (**d**) PG screw, and (**e**) close-up view of PG screw, where it is possible to observe greater damage to PTFE tape, with more tears and cuts.

After loosening the six nCL samples, the SEM analysis revealed that in the GG sample, GapSeal^®^ can easily be seen to cover a good portion of the screw, especially the internal areas of the turns. In the GP sample, we can see that the PTFE tape is unevenly distributed along the screw. In sample 1, there is no PTFE tape in the apical area, but in sample 2 there is. Tears/cuts in the PTFE tape were found. After loosening the six CL samples, in the GG, comparing the screw images from the two samples, we see the presence of GapSeal^®^, which is distributed unequally between the samples: sample 1 has less than sample 2. In the GP sample, we can see that the PTFE tape is much more damaged, with many more tears when compared to the nCL samples, and there are several areas of the screw without PTFE tape. All analyses show that both coatings are found in the innermost areas of the thread (Figure 5 and Figure 6).

## 4. Discussion

This in vitro study aimed to assess the influence of the application of two coating materials (PTFE tape and silicone sealing gel—GapSeal^®^) on prosthetic screws to prevent screw loosening. The effectiveness was assessed by analyzing the screw preload and the RTV with and without a CL. The results showed that wrapping the screw with PTFE tape significantly reduced the preload, regardless of the presence of a CL. The results revealed that wrapping the prosthetic screw with PTFE tape in Group 1 (nCL) significantly reduced the RTV, but in the presence of a CL under oral conditions, the PTFE tape significantly increased the RTV. Micro-CT analysis shows that coating with PTFE tape and GapSeal^®^ did not interfere with the interface between the implant and the abutment, and no microgaps were found. The SEM analyses show that both coatings are found in the innermost areas of the thread, the PTFE tape in the samples subjected to a CL present more tears/cuts, and the GapSeal^®^ is less evenly distributed. Those two materials were specifically chosen because they can be applied by the dentist when a screw comes loose.

### 4.1. Preload Analysis

The Group 1 (nCL) results revealed that the preload values in the PG were significantly lower than those in the CG and GG. Therefore, the null hypothesis was rejected for the group in which PTFE tape was used to wrap the screws. Previous studies have shown that preload is lost due to the settling effect, which is directly related to the roughness of the screw surface. Surface roughness refers to microscopic texture or irregularities on a surface, and no matter how well-machined screws are, they still present some irregularities, which will cause a loss of preload while tightening. Considering that the screw will need more energy to overcome this microroughness, increasing roughness will increase the coefficient of friction because more asperities come into contact and create more resistance. [4,8]. To this end, researchers have begun to test the application of various coatings that act as lubricants, which will reduce the coefficient of friction during tightening, making it possible to obtain a greater preload during the first round of tightening. In our study, the ability to use PTFE tape and a sealing silicone gel, GapSeal^®^, as lubricant coatings was tested, allowing us to compare screws with these coatings with uncoated screws.

Our results revealed that coating the screw with PTFE tape significantly reduced preloads in Group 1 (nCL) and Group 2 (CL), which did not meet the objective of reducing the coefficient of friction to obtain a greater preload, even if the coefficient of friction was not measured directly. These results are aligned with those of another study [28]. It is possible that the application of PTFE tape leads to greater instability during bolt tightening, explaining why the preload values are lower in this group than in the other groups. Chen et al. [29] also tested PTFE as a screw coating, and they found a significant reduction on the coefficient of friction and a significantly higher clamping force (preload) on the screw joint. These results differ from our findings, which can be explained by differences in methodology, like the fact that the authors of the previous study did not analyze the screw preload, and the PTFE was applied via thermal spraying. The thickness of the material may have also impacted the results; in our study, the PTFE tape had a thickness of 0.15 mm, whereas Chen et al. [29] tested two thicknesses, 0.03 mm and 0.06 mm, and the best results were obtained for the group with the thinner coating. They explained that these results are due to the more intimate contact of thicker coatings compared with thinner ones.

In five previous studies [18,28,30,31,32], the application of a sealing silicone gel was tested using two brands: KieroSeal^®^ and GapSeal^®^. Among these studies, the screw preload was evaluated in only one work [28], the results of which align with ours, as they reported that GapSeal^®^ did not influence the preload. Biscoping et al. [30] used KieroSeal^®^, and the preload values they obtained were all slightly lower; however, their statistical analysis was incomplete, and the significance of the results in relation to the coatings has not been reported. Rathe et al. [31] evaluated the screw-induced preload after coating the screws with KieroSeal^®^ and applying different torques. They reported similar results, namely, that there were no significant differences among some of the groups; however, in the groups with lower torques, the preload was significantly lower. This could be explained because in this study, the preload induced by the screw at the joint was analyzed instead of the screw preload, furthermore KieroSeal^®^ has different properties than GapSeal^®^.

Different thicknesses of PTFE can lead to different screw behaviors during screw tightening. The greater thickness of the PTFE and the way it is presented, (which, in the case of PTFE tape, makes it difficult to ensure that it is equally well distributed throughout the screw) could lead to excessive slippage during screw tightening, causing instability and the need to expend force to overcome these adversities. The SEM images confirm this statement, in the two samples from the PG, we can see that the distribution of the PTFE tape along the screw differs from one sample to the next: one screw is almost entirely covered with PTFE tape and the other has areas without PTFE. 

### 4.2. Micro-CT Analysis

The different coatings applied at a clinical appointment differ in the type and form of presentation: liquid, gel, solid. Therefore, there are different viscosity of the various mediums: blood 3.5-5.5 cP, 22 saliva 2.5-6 cP, 23 fluoride mouthwash, >500 cP, 24 and CHX mouthwash > 100 cP, 25 and gel > 200 cP whereas that of air is about 0 [17]. The solid form corresponds to the PTFE tape which has different thickness. To ensure that this coating does not interfere with the fit of the abutment to the implant, one author analyzed the samples in a stereomicroscope [19]. This tool is very useful for analyzing small areas such as the fit between the abutment and the column, although it can only do so in the part that is visible. Microgaps at the implant–abutment connection interface should be avoided for abutment stability. Microgap-related problems are both biological (like bacterial colonization) and mechanical (micromovements will lead to the fatigue of the abutment screw, loss of preload among others) [33]. 

Recently, some studies have been carried out in the field of dental medicine, showing the applicability of Micro-CT [34,35,36]. This is a non-destructive imaging technique based on X-rays that allows the rapid digitization of samples in three dimensions, and it has the ability to visualize the interior and exterior characteristics of a sample [37]. 

Our results show that the application of the coatings did not interfere with the fit between the abutment and the implant: the amount of gel applied was not excessive enough for it to leak into this area, and the PTFE tape was carefully applied so that it did not move into this area. To date, we have not found any other article that has evaluated whether the application of coating interfered with the external and/or internal adjustment of the abutment/implant complex.

### 4.3. RTV Analysis

Although coatings could increase the preload, it is also important that the preload remains stable instead of being lost quickly, especially under masticatory stress. Therefore, we also assessed the effect of coating on the RTV in nCL and CL conditions.

The results revealed that the RTV in the PG was significantly lower than those in the CG and GG under nCL conditions: almost 49% of the torque was lost. In contrast, the RTV in the PG was significantly greater than those in the other groups under CL conditions. Therefore, the null hypothesis was rejected for the group in which PTFE tape was used as a coating. Given the different results obtained under the two conditions, we analyzed them separately.

For Group 1 (nCL), some of these results are in line with those of Coelho et al. [28], particularly the results for the PG; they also reported that the RTV was significantly lower. However, the GG results differ from ours, as the authors found that coating the screws with GapSeal^®^ significantly raised the RTV. The fact that the amount of silicone gel placed inside the implant was not quantified could have led to different results. A smaller amount of gel could have resulted in the gel not covering the entire surface of the screw threads. SEM images confirm this; in two samples from the GG, we can see that the amount of silicone gel sealing varies between the samples, with one being practically covered in GapSeal^®^ and the other having a much lower amount. Even so, it may have happened that when the screw was removed, some of the gel remained inside the implant instead of sticking to the screw. The only study [30] analyzing the influence of a sealing silicone gel in nCL conditions recorded a higher RTV in samples coated with silicone gel; however, since these authors did not perform a complete statistical analysis, we cannot compare our results with their findings. With respect to the PG, the only study in which PTFE tape was used [19] reported different results from our findings, including a significantly higher RTV for the samples using PTFE tape. The main difference was the thickness; we used 0.15 mm thick PTFE tape, whereas 0.30 mm thick PTFE tape was used in this previous study. It is possible that the thicker coating made the thread contact more intimate or the surface stress distribution of the thread was paired more uniformly. However, they evaluated the RTV after applying the CL, and this difference in methodology makes it difficult to compare the results. Elias et al. [38] reported similar results with a significantly lower RTV, whereas Chen et al. [29] reported a significantly higher RTV; however, these authors did not use PTFE tape but applied a coating via thermal spraying, which may explain the difference in results.

The results for Group 2 (CL) were surprising, considering the Group 1 (nCL) results for the PG in the RTV. Under CL conditions, we found that the RTV was significantly greater in the PG, which is in line with other studies [19,29]. Pure PTFE is known for its low friction and high wear resistance, which are attributed to its low intermolecular cohesion and its ability to form a thin transfer film on its sliding counterpart. It seems that the sliding environment affects wear more than friction does, and the impact of moisture on both the coefficient of friction and specific wear rate has been shown to reach approximately 40% [39]. Under CL conditions, there are sliding movements between the screw and the implant, which likely cause the PTFE to wear and deform or even tear. The micro-deformation of the PTFE tape could then fill the voids in the rough regions between the screw and implant walls, leading to mechanical interlocking and creating a unique structure that absorbs vibrations and shocks. The SEM images confirm this statement: in the two samples from the PG after the CL, we can see that the PTFE is more damaged with more tears and cuts, and the tape is located on the inside of the screw turns. However, our study differs from previous works in terms of one key result: the mean RTV in the PG was higher than the mean preload value. Thus, in addition to the settling effect and the influence of the CL on the reduction in the preload value, the RTV increased. While GC and GG samples demonstrated preload losses of around 18% and 12.6%, respectively, PG had an increase in preload of 1.88% instead. Our study is the only work in which a PTFE tape was applied to the screw and the tests were carried out in a warm and humid environment, simulating the oral environment. Although PTFE is considered a hydrophobic material, when exposed to high humidity levels and warm temperatures over a long period, water molecules and the PTFE may interact, causing the PTFE to expand slightly, filling the existing microgaps better and more compactly under these conditions. Widziewicz-Rzońca and Tytła [40] revealed that despite the excellent hydrophobic properties of PTFE, a small amount of water is absorbed by this material. Coating a screw with a product that expands will increase the force needed to loosen it. We believe that RTV surpassing the tightening torque will not have clinical consequences like screw fracture, or damage the implant’s internal threading. 

Additionally, the applicability of a sealing gel as a screw coating was tested because it was hypothesized that the high viscosity of the gel could act as a protective layer, helping to reduce the impact of the external load, preventing the reduction in the torque [18]. Although the RTV was greater in the GG than in the CG, this difference was not significant. Similar results were reported by Yu et al. [18], who used the same sealing silicone gel; however, in their study, it significantly reduced the percentage of torque loss. In contrast, Ozdiler et al. [32] reported significantly lower RTVs for screws coated with KieroSeal^®^, and the difference in the results could be related to the shrinkage behavior of this specific sealing silicone gel, which the initial size changed due to polymerization, whereas GapSeal^®^ is a highly viscous material that never hardens, thereby preventing shrinkage-related gaps.

Although we attempted to simulate the oral cavity as closely as possible, the experiments were still performed in vitro, which limits the extrapolation of the results to real-world settings. The placement of PTFE tape is difficult to standardize, even if it is always applied by the same operator. This material is very sensitive to handling; therefore, we cannot guarantee that the tape was distributed uniformly and in the same manner for all the screw samples. Considering the properties of this material and the good results obtained, it would be interesting to have a PTFE spray, so that it could be applied by the dentist during a dental appointment. Additionally, the amount of GapSeal^®^ applied could be quantified to reduce variability among the samples; it would be particularly useful, for example, to be able to know the ideal amount of GapSeal^®^ based on the size of the implant. In a future study, it would be useful to test these procedures on a human patient to determine how these materials actually behave.

## 5. Conclusions

Within the scope of this study, the following conclusions can be drawn:

- The application of PTFE tape on the prosthetic screw significantly reduces the preload.
- The application of GapSeal^®^ and PTFE tape did not create microgaps between the implant and the abutment.
- Under nCL conditions, wrapping the prosthetic screw with PTFE tape significantly reduces the RTV, almost 49% of the preload was lost.
- Under CL conditions in a warm and humid environment simulating the oral cavity, wrapping the prosthetic screw with PTFE tape results in a significantly greater RTV, and in addition to not having the expected loss of preload, there was still a gain of around 1.8%.
- SEM analysis showed that the coatings flow into the innermost areas of the screw threads, corresponding to the hypothetical free spaces between the implant and the screw. In the case of PTFE tape, as it expands, this complex becomes a single unique structure, making it more resistant to loosening.

Within the limitations of this in vitro study, we can conclude that coating the prosthetic screw with PTFE could prevent the screw from loosening. However, more clinical trials should be conducted, quantifying the GapSeal^®^, testing different PTFE tape thickness and using different implant connections. Also, it would be interesting to perform a profilometry to assess changes in the screw/coating interface after testing.

## Figures and Tables

**Table 1:** Factorial plan.

Total Sample	Groups	Subgroups	Screw Tightening and Preload Register	Micro-CT Analysis	Untightening and RTV Register	CL	Untightening and RTV Register	SEM Analysis
*n* = 90	Group 1 *n* = 45	CG *n* = 15	*n* = 15	*n* = 2	*n* = 15	X	X	*n* = 2
		GG *n* = 15	*n* = 15	*n* = 2	*n* = 15	X	X	*n* = 2
		GP *n* = 15	*n* = 15	*n* = 2	*n* = 15	X	X	*n* = 2
	Group 2 *n* = 45	CG *n* = 15	*n* = 15	X	X	*n* = 15	*n* = 15	*n* = 2
		GP *n* = 15	*n* = 15	X	X	*n* = 15	*n* = 15	*n* = 2
		PG *n* = 15	*n* = 15	X	X	*n* = 15	*n* = 15	*n* = 2
Total	*n* = 90	*n* = 90	*n* = 90	*n* = 6	*n* = 45	*n* = 45	*n* = 45	*n* = 12

X—Not applicable.

**Table 2:** Initial comparisons of preload and RTV in the Group 1 (nCL).

	CG (*n* = 15) M (SD)	GG (*n* = 15) M (SD)	PG (*n* = 15) M (SD)	ANOVA
Preload (Ncm)	30.95 (1.00)	31.19 (1.22)	29.92 (0.76)	F_(2, 42)_ = 6.56 (*p* = 0.003), η^2^=0.24
RTV (Ncm)	27.98 (1.20)	28.48 (1.47)	15.30 (1.21)	F_(2, 42)_ = 496.50 (*p* < 0.001), η^2^=0.94

Tukey HSD for preload: CG vs. GG (*p* = 0.787), CG vs. PG (*p* = 0.022), and PG vs. GG (*p* = 0.004); Tukey’s HSD for RTV: CG vs. GG (*p* = 0.547), CG vs. PG (*p* < 0.001), and PG vs. GG (*p* < 0.001).

**Table 3:** Initial comparisons of the preload and RTV for Group 2 (CL).

	CG (*n* = 15) M (SD)	GG (*n* = 15) M (SD)	PG (*n* = 15) M (SD)	ANOVA
Preload (Ncm)	31.72 (1.16)	31.42 (0.98)	30.29 (0.83)	F_(2, 42)_=8.51 (*p* < 0.001), η^2^ = 0.29
RTV (Ncm)	26.00 (2.00)	27.44 (3.76)	31.50 (0.80)	F_(2, 42)_=19.43 (*p* < 0.001), η^2^ = 0.48

Tukey HSD for preload: CG vs. GG (*p* = 0.696), CG vs. PG (*p* < 0.001), and PG vs. GG (*p* = 0.009); Tukey HSD for RTV: CG vs. GG (*p* = 0.268), CG vs. PG (*p* < 0.001), and PG vs. GG (*p* < 0.001).

## Data Availability

The original contributions presented in this study are included in the article material. Further inquiries can be directed to the corresponding author.
